# Supplementary material for: Toggle-like current-induced Bloch point dynamics of 3D skyrmion strings in a room temperature nanowire
Source: Nat Commun. 2022 Jun 24;13:3630. doi: 10.1038/s41467-022-31335-y (PMC9232487; doi:10.1038/s41467-022-31335-y)
Supplement: Supplementary file 1 — Supplementary Information [file 41467_2022_31335_MOESM1_ESM.pdf]

Supplementary Information: Toggle-like current-induced Bloch point dynamics of 3D skyrmion strings in a room temperature nanowire

M. T. Birch[1], D. Cortés-Ortuño[2], K. Litzius[1], S. Wintz[1,3], F. Schulz[1], M. Weigand[3], A. Štefančič[4,5], D. A. Mayoh[4], G. Balakrishnan[4], P. D. Hatton[6], G. Schütz[1]

[1]*Max Planck Institute for Intelligent Systems, 70569 Stuttgart, Germany*

[2]*Department of Earth Sciences, Utrecht University, 3584 CB Utrecht, The Netherlands*

[3]*Helmholtz-Zentrum Berlin für Materialien und Energie GmbH, 12489 Berlin, Germany*

[4]*Department of Physics, University of Warwick, Coventry, CV4 7AL, UK*

[5]*Electrochemistry Laboratory, Paul Scherrer Institut, CH-5232 Villigen PSI, Switzerland*

[6]*Department of Physics, Durham University, Durham DH1 3LE, UK*

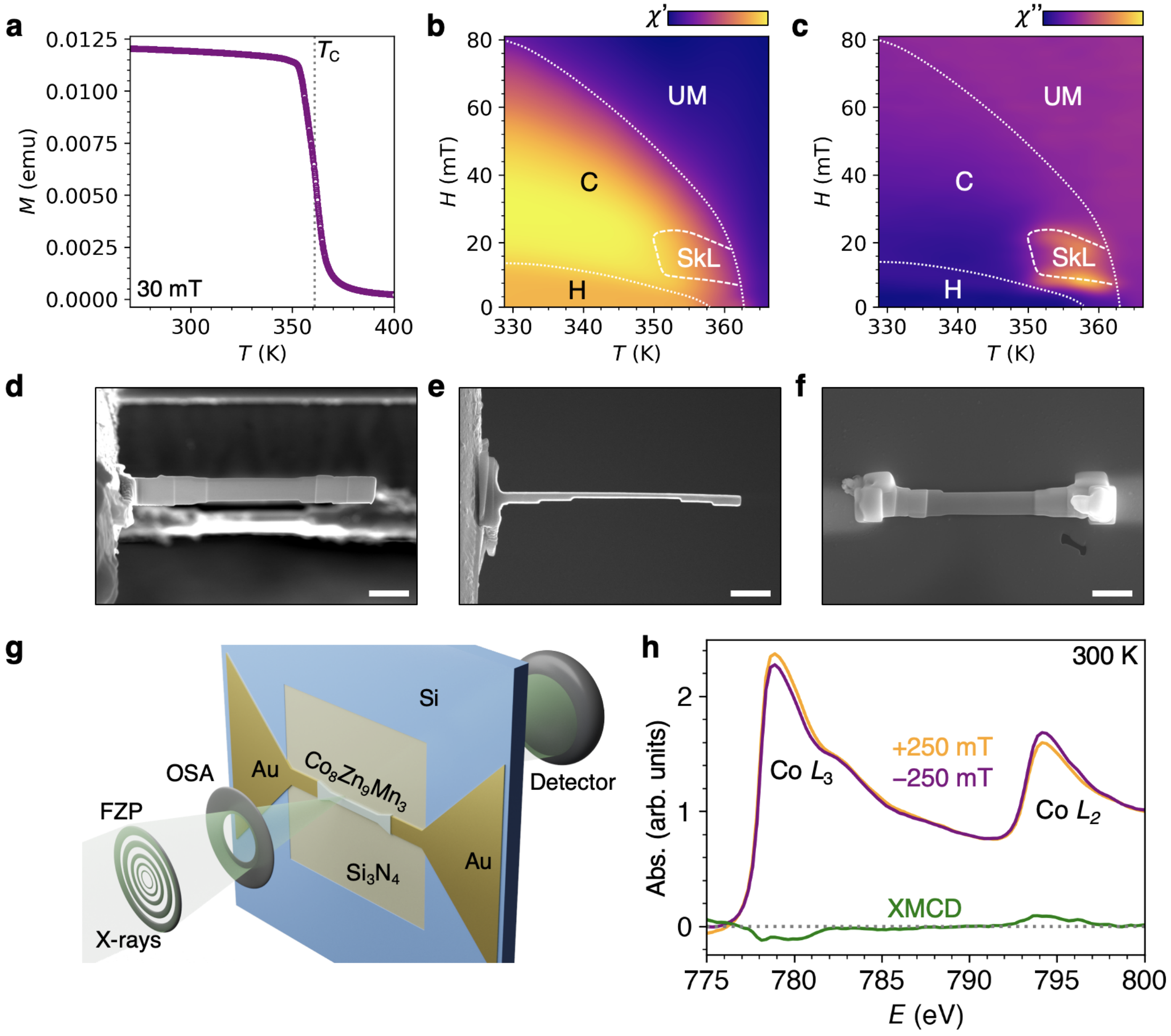

Figure S1 | **Bulk sample characterisation and nanowire fabrication.** **a**, Magnetisation, M, measured as a function of decreasing temperature, T, on the bulk $Co_8Zn_9Mn_3$ single crystal sample under an applied magnetic field of 30 mT. The measured T_C is ~361 K. **b**,**c**, The real and imaginary components of the AC susceptibility, χ' and χ'' respectively, measured with the single crystal, as a function of increasing field B at each temperature. The boundaries separating the helical (H), conical (C), uniformly magnetised (UM) and equilibrium skyrmion lattice (SkL) states are indicated. **d**-**f**, Scanning electron microscopy images of the nanowire sample as it was fabricated (d,e), and after being fixed to the gold (Au) contacts on the Si_3N_4 membrane (f). The scale bar is 1 µm. **g**, Schematic illustration of the scanning transmission x-ray microscopy technique, where the incoming x-rays are focused to a ~20 nm spot size by a Fresnel zone plate (FZP) and order selecting aperture (OSA). Transmitted photons are counted by the photodiode detector. **h**, The absorption of the nanowire sample measured as a function of x-ray energy E across the Co L_3 and L_2 edges. By measuring for applied out-of-plane magnetic fields of ±250 mT (orange and yellow), and subtracting the two datasets, the x-ray magnetic circular dichroism (XMCD) signal is revealed (green).

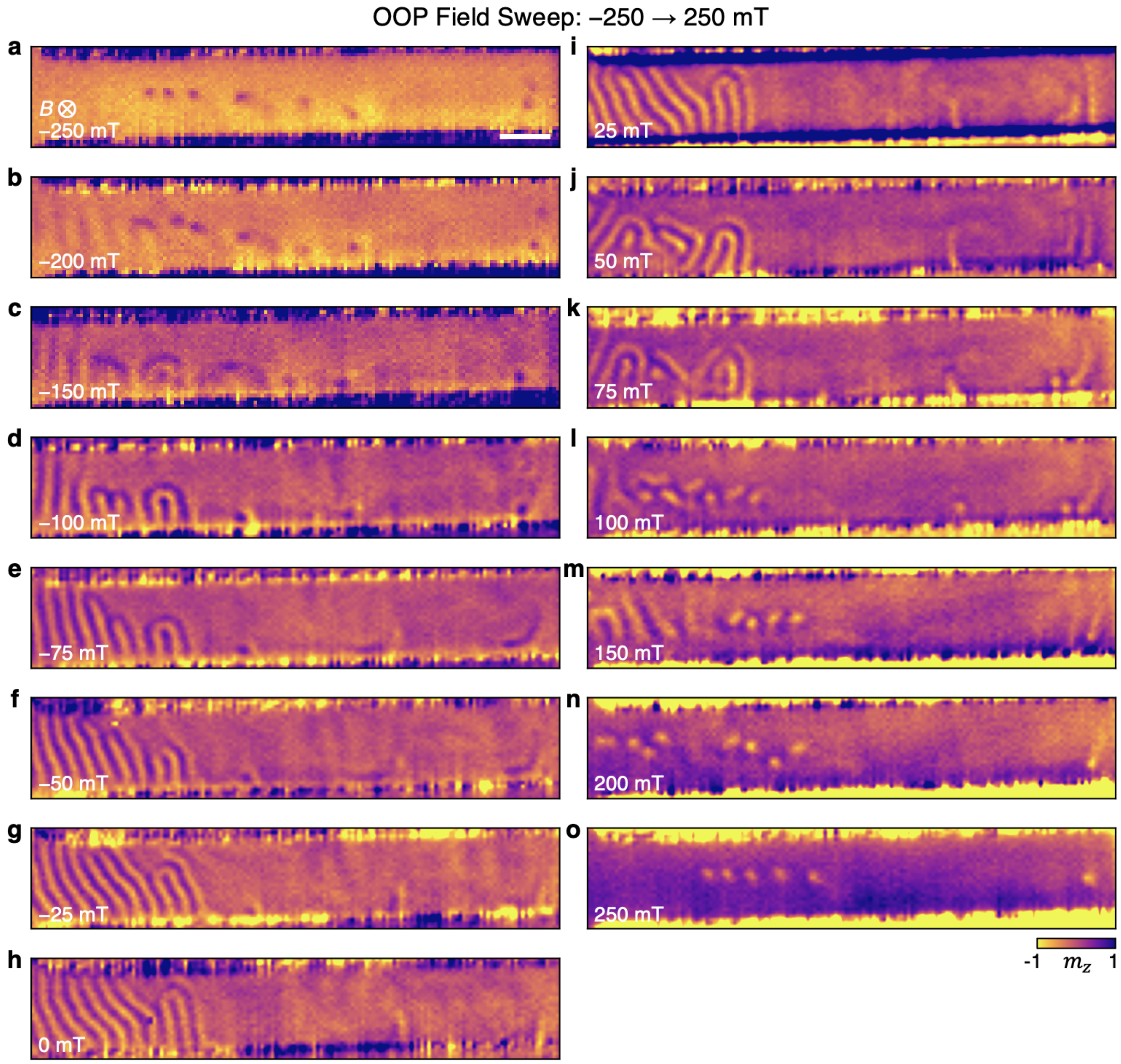

Figure S2 | **Out-of-plane magnetic field sweep. a-o**, Scanning transmission x-ray microscopy images of the $Co_8Zn_9Mn_3$ nanowire sample, acquired at 300 K as a function of increasing out-of-plane field starting from -250 mT. The colourmap indicates the out-of-plane magnetisation m_z. This data was utilised to create the single temperature phase diagram in Fig. 2a of the main text. The scale bar is 500 nm.

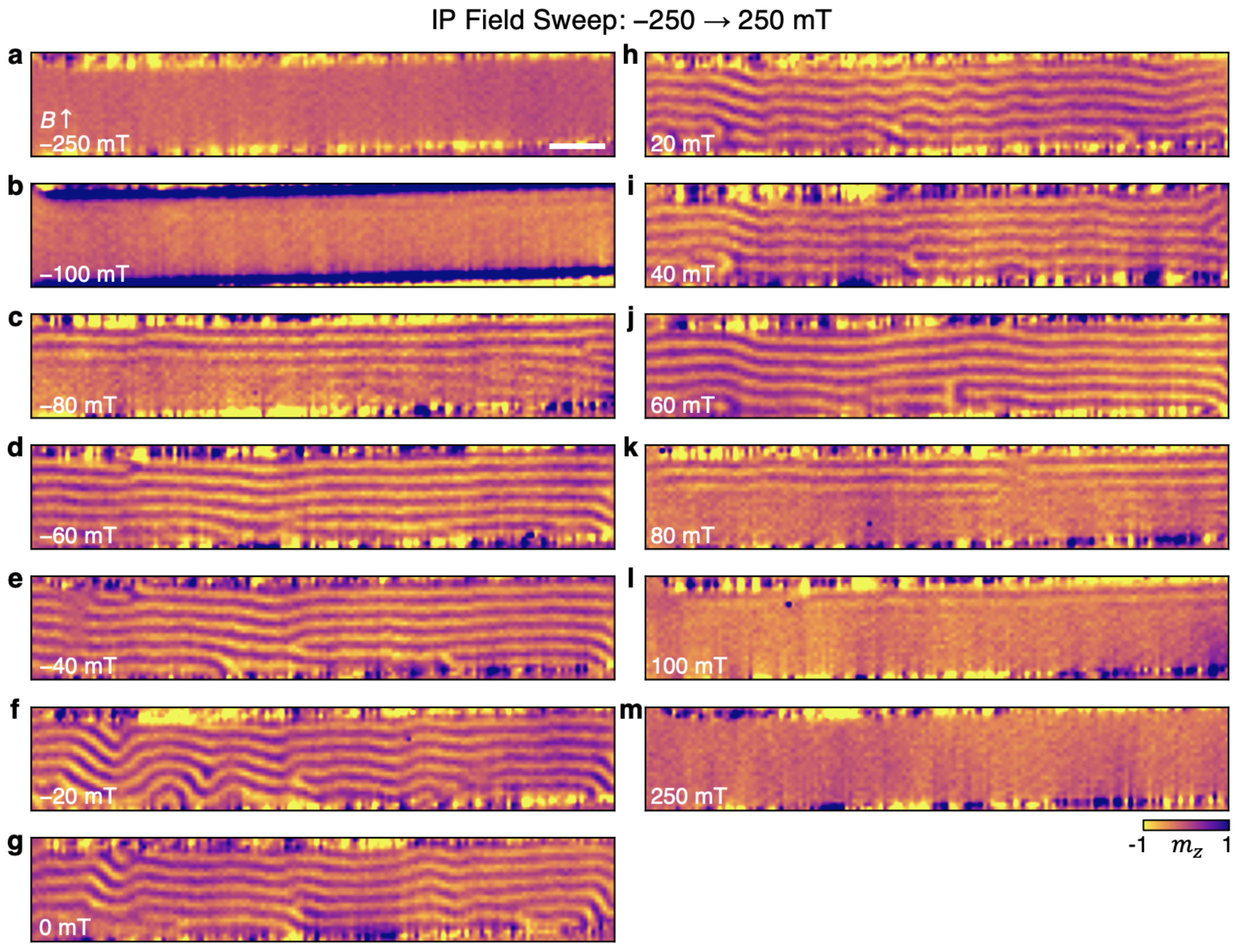

Figure S3 | **In-plane magnetic field sweep. a-m**, Scanning transmission x-ray microscopy images of the nanowire sample, acquired at 300 K as a function of increasing in-plane field starting from -250 mT. The colourmap indicates the out-of-plane magnetisation m_z. This data was utilised to create the single temperature phase diagram in Fig. 2b of the main text. The scale bar is 500 nm.

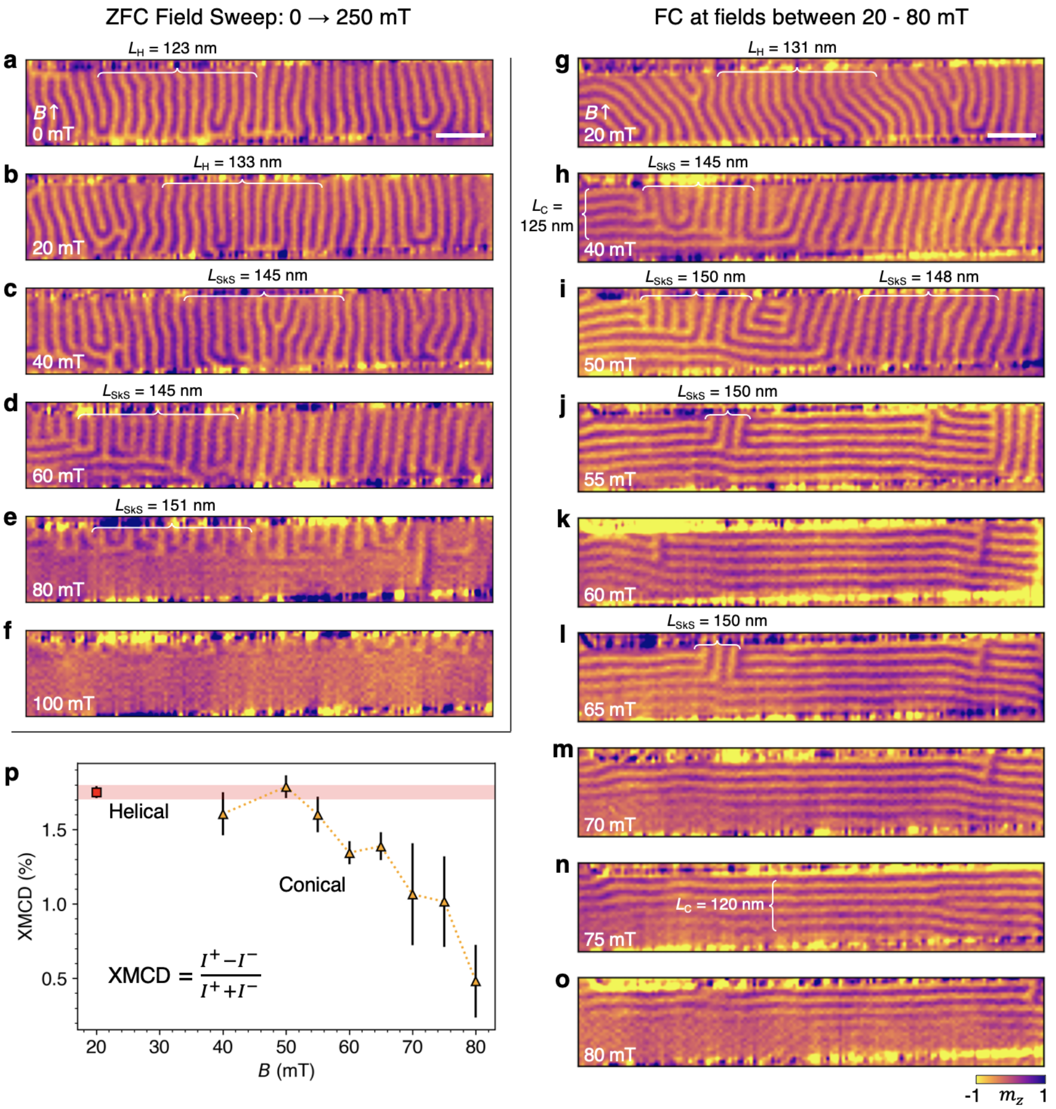

Figure S4 | **Zero field-cooled and field-cooled data. a-f**, Scanning transmission x-ray microscopy images of the nanowire sample, acquired at 300 K as a function of increasing in-plane field starting from 0 mT after an initial zero field-cooling (ZFC) from above T_C. The images reveal the formation of skyrmion strings (SkS) in c from the initial helical state in a, as indicated by the change in spacing of the vertical contrast structures, labelled as either the helical winding length L_H, conical winding length L_C, or SkS separation L_{SkS}. **g-o**, Images of the nanowire acquired upon field-cooling (FC) from above T_C at a range of applied in-plane fields between 20 and 80 mT. The presence of SkSs was identified by the vertical contrast spacing. This data was utilised to create the single temperature phase diagram in Fig. 2c of the main text. The colourmap indicates the out-of-plane magnetisation m_z. The scale bar is 500 nm. **p**, the maximum x-ray magnetic circular dichroism (XMCD) contrast, calculated as shown in the equation where I^+ and I^- are the intensities of the right and left circularly polarised light, of the helical (red) and conical (orange) states in g-h, plotted as a function of the applied in-plane field B. The data shows the reduction in XMCD contrast of the conical state as the spins tilt towards the field direction. The error bars indicate the standard error calculated when averaging over multiple winding lengths.

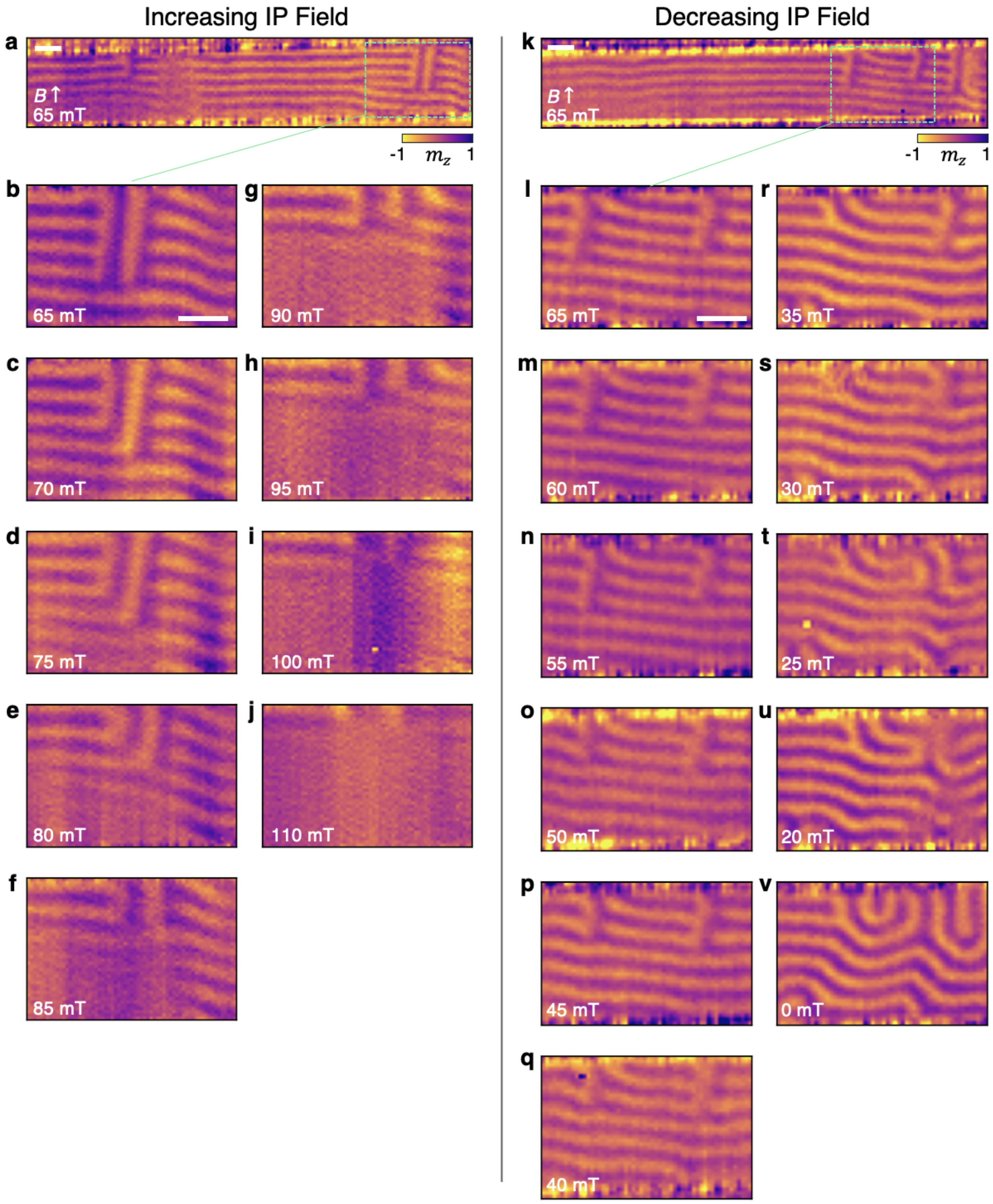

Figure S5 | **Skyrmion string stability against applied magnetic field. a**, Scanning transmission x-ray microscopy image of the nanowire sample at at 300 K, showing the formation of the two adjacent SkSs state. **b-j**, Images showing the annihilation of the SkS state as a function of increasing in-plane (IP) applied magnetic field, transforming the SkSs into chiral bobber surface states. **k**, Image of the nanowire, showing the formation of the separated SkS state. **l-v**, Images showing the annihilation of the two separated SkSs state as a function of decreasing IP applied magnetic field, showing the transformation of SkSs into the helical state at 0 mT. The colourmap indicates the out-of-plane magnetisation m_z. All scale bars are 250 nm.

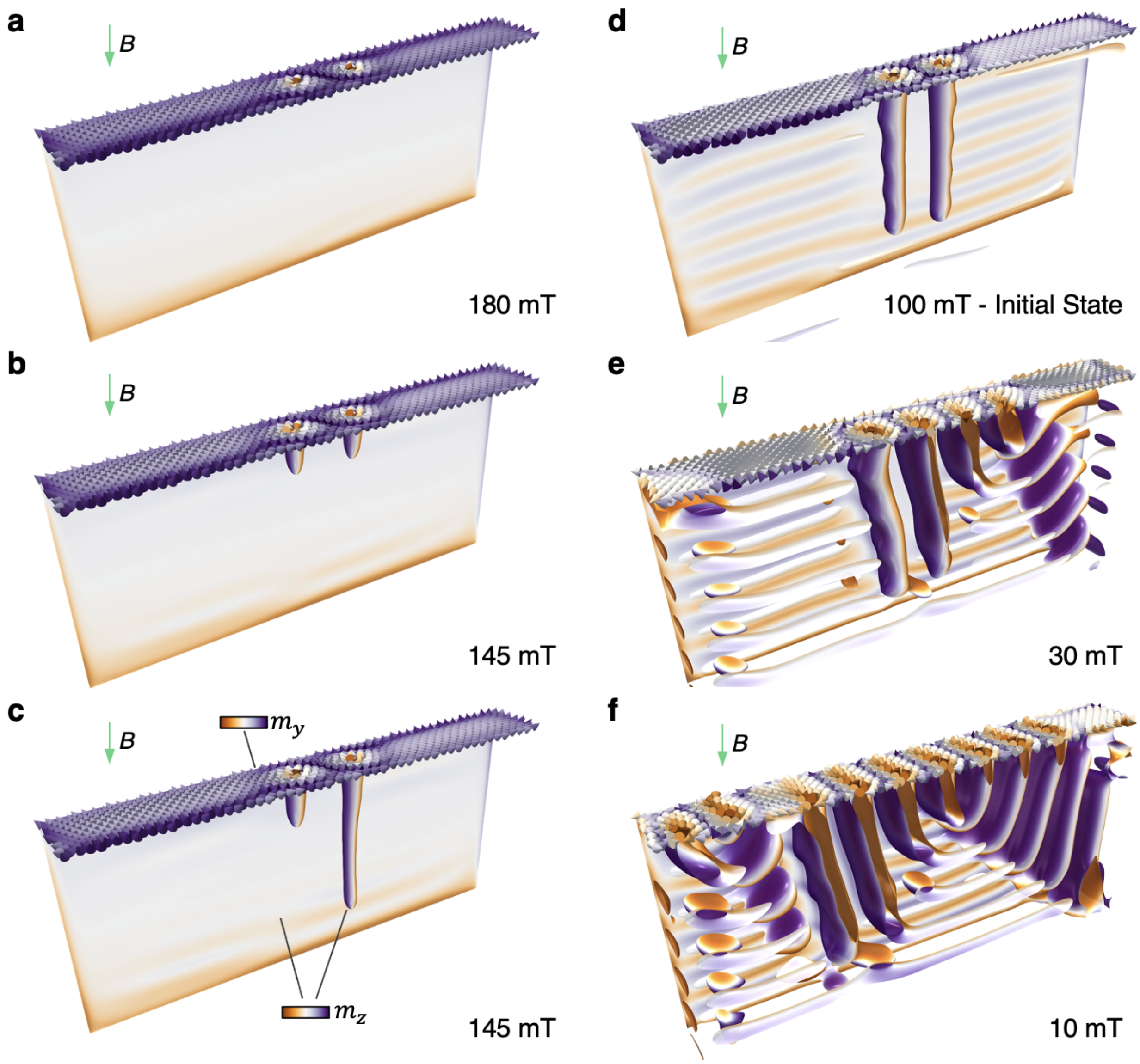

Figure S6 | **Visualisations of the simulated adjacent skyrmion string state. a-f**, Three dimensional visualisations of the magnetic configurations simulated for the two adjacent skyrmion string (SkS) state. The images were achieved by plotting contours showing regions where the local value of $m_y = 0$. The state initialised and relaxed at 100 mT is shown in d. From this starting point, the applied magnetic field was increased or decreased, and the system was once again relaxed, resulting in the configurations shown. For higher applied fields, the SkSs unwind along their length to form the chiral bobber state at the surface of the sample. The colour maps show the local orientation of the magnetisation, as indicated by the colour bars.

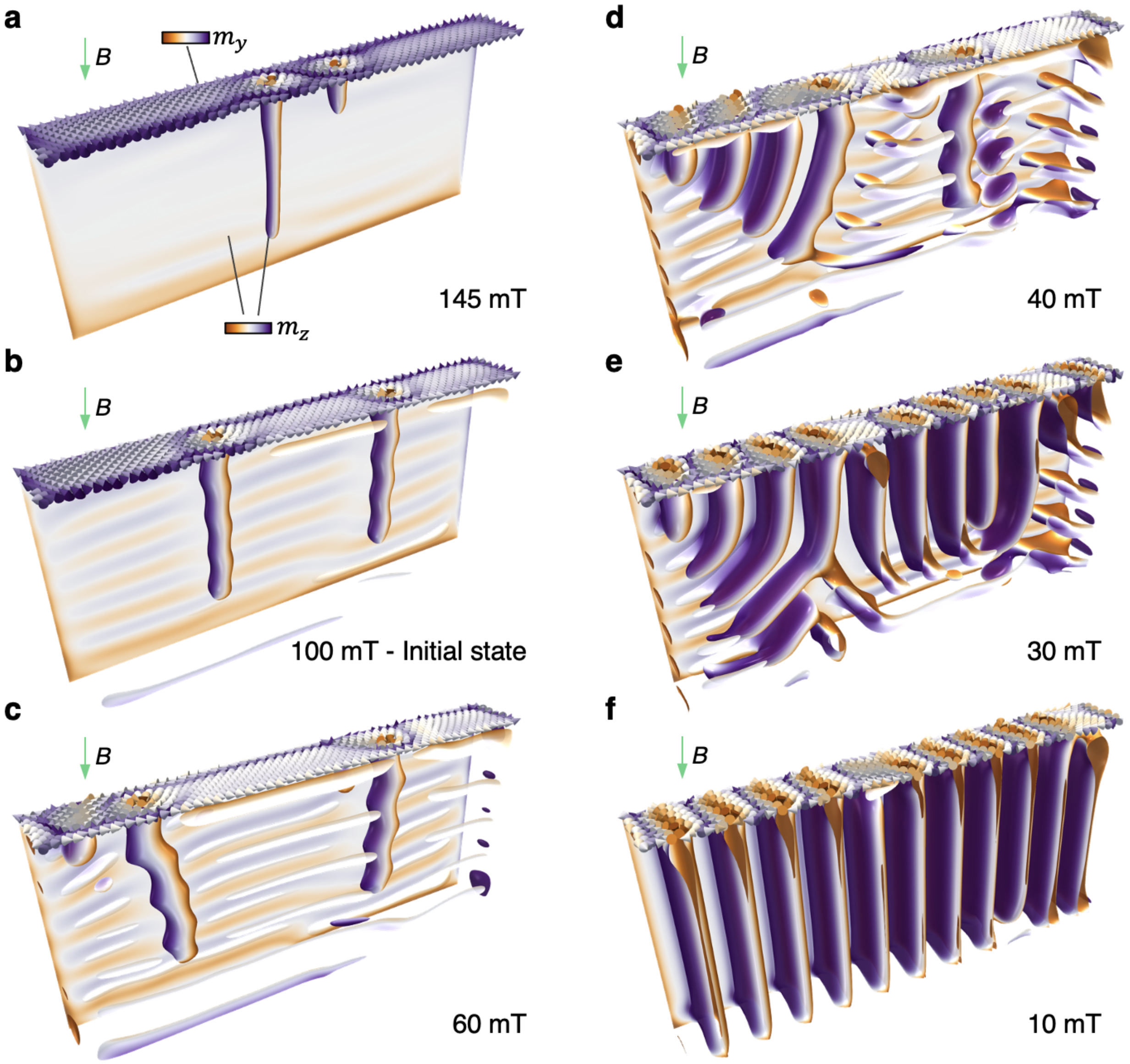

Figure S7 | **Visualisations of the simulated separated skyrmion string field sweep. a-f**, Three dimensional visualisations of the magnetic configurations simulated for the two separated skyrmion string (SkS) state. The images were achieved by plotting contours showing regions where the local value of $m_y = 0$. The state initialised and relaxed at 100 mT is shown in b. From this starting point, the applied magnetic field was increased or decreased, and the system was once again relaxed, resulting in the configurations shown. For lower applied fields, the SkSs transform into the helical domain state. The colour maps label the local orientation of the magnetisation, as indicated by the colour bars.

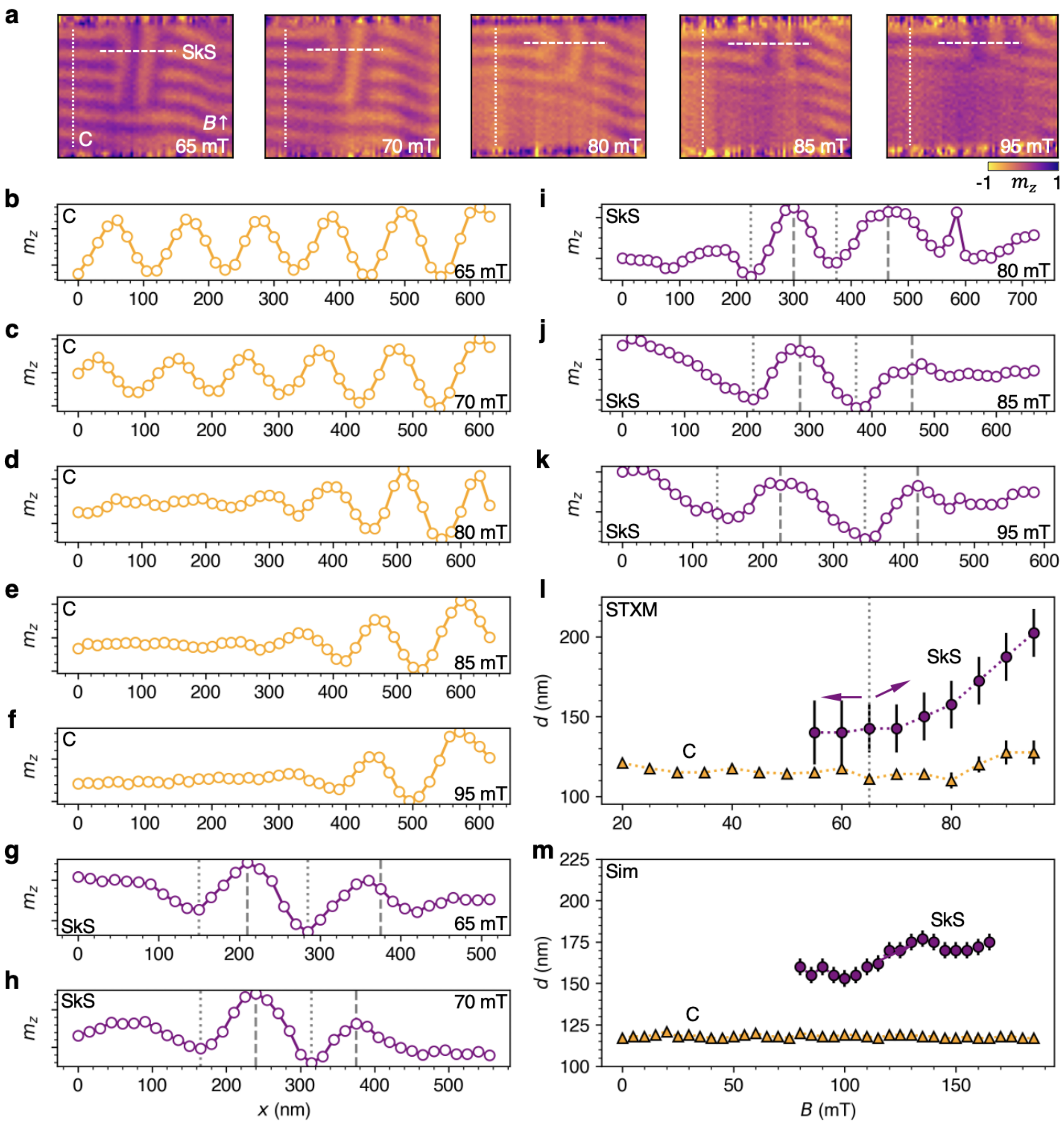

Figure S8 | **Conical and skyrmion string spacing determination.** **a**, Scanning transmission x-ray microscopy (STXM) images of the two adjacent skyrmion string (SkS) state as a function of increasing applied in-plane magnetic field at 300 K. The dotted and dashed lines indicate profiles of the magnetisation component m_z taken through the conical (C) and SkS states, respectively. **b**-**f**, The m_z profiles for the C state from the STXM images in a. **g**-**k**, The m_z profiles for the SkS state from the STXM images in a. **l**, The average spacing of SkS (purple circles) and C (yellow triangles) states plotted as a function of the applied in-plane magnetic field, after being extracted from the data in b-k. The dashed line and arrows indicate the initial field upon field cooling, and the direction of the magnetic field change. Error bars indicate the error calculated due to the resolution limit, and the averaged number of measured SkS/C spacings. **m**, The average spacing of SkS (purple circles) and C (yellow triangles) states plotted as a function of the applied in-plane magnetic field, recovered from m_z profiles of the micromagnetic simulations. Error bars indicate the 5 nm resolution limit of the simulated system.

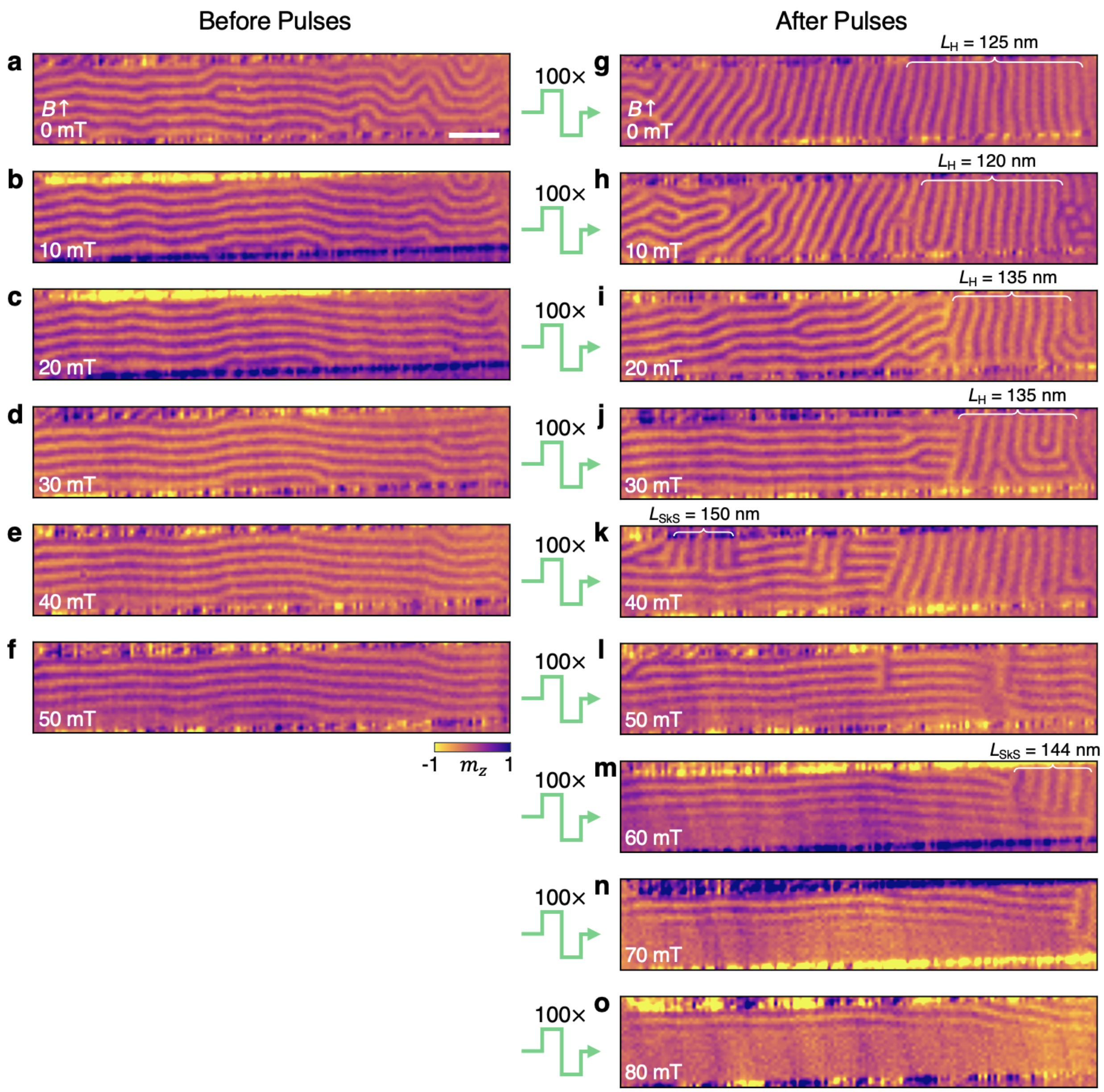

Figure S9 | **Nucleating skyrmion strings with current pulses. a-f**, Scanning transmission x-ray microscopy images of the nanowire sample at 300 K. Images show the formation of the conical state after resetting the sample by saturating the magnetisation in-plane by applying a -250 mT field, and subsequently applying the field specified in each image. **g-o**, Images of the nanowire, showing the magnetic state achieved after applying 100 bipolar current pulses of duration 30 ns and with a current density of 6×10^{10} A/m^2, as indicated by the green arrows. For low applied fields, this results in the formation of the helical state, or even out-of-plane skyrmions in h. However, for fields between 40 to 70 mT, skyrmion strings (SkS) are nucleated. The identity of the SkSs is verified by comparing their spacing to the winding length of the surrounding conical state as indicated by the labelled measurements of the helical winding length L_{H}, or SkS separation L_{SkS}. The colourmap indicates the out-of-plane magnetisation m_z. The scale bar is 500 nm.

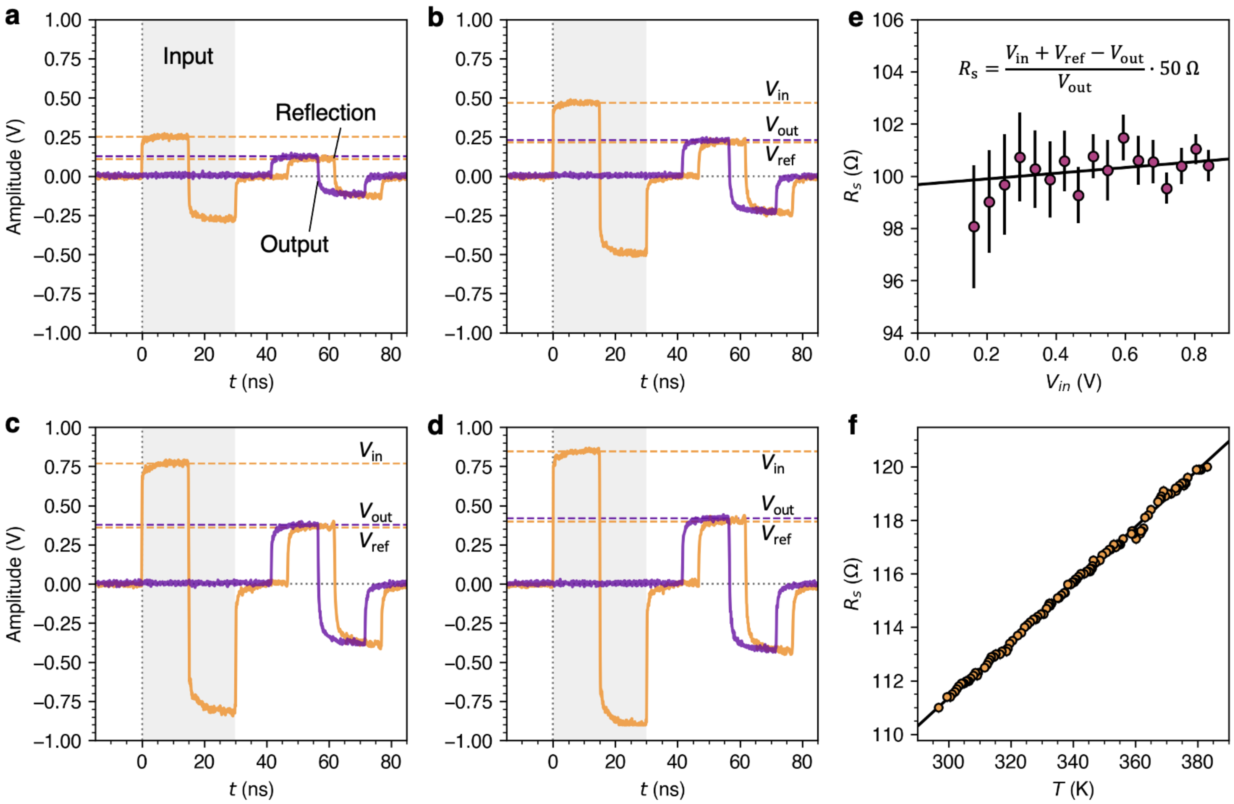

Figure S10 | **Joule heating estimation and resistance-temperature calibration. a-d**, Example 30 ns bipolar pulse traces, showing the input V_{in} and reflected V_{ref} signal in the first channel (orange), and the output signal V_{out} transmitted through the sample in the second channel (purple), for different input current densities. **e**, The measured sample resistance R_{s} as a function of V_{in}, following calculations using the equation shown. Error bars indicate the standard error determined by measurement of 10 or more pulses at each V_{in}. **f**, A measurement of the sample resistance, R_{s} as a function of the temperature, T, used as a calibration for the Joule heating estimate.
